# New strategy in hemorrhagic gastric cancer: A case report of complete pathological remission after neoadjuvant chemotherapy

**DOI:** 10.1097/MD.0000000000032789

**Published:** 2023-02-10

**Authors:** Yuhang Zhou, Yuchen Zhou, Xiaojun Lin, Shengtao Lin, Weihua Li

**Affiliations:** a Shengli Clinical Medical College of Fujian Medical University, Fuzhou, China; b Department of Surgical Oncology, Fujian Provincial Hospital, Fuzhou, China; c The First Affiliated Hospital of Fujian Medical University, Fuzhou, China.

**Keywords:** case report, complete pathological remission, hemorrhagic gastric cancer, locally advanced gastric cancer, neoadjuvant chemotherapy

## Abstract

Strategy for hemorrhagic gastric cancer should both handle the potential life-threatening situation caused by bleeding and increase probability of long-term survival. For hemorrhagic patients with locally advanced gastric cancer, surgical resection is always the preferred option for the reason that it eliminates both the tumor and risk of rebleeding. However, the long-term survival after resection is still unsatisfactory.

**Patient concerns:**

Here, we report a patient with hemorrhagic locally advanced gastric cancer achieved pathological complete response after neoadjuvant chemotherapy.

**Diagnoses:**

In this case, a 58-year-old man presenting with gastrointestinal hemorrhage and hemodynamic instability was admitted to the emergency department. Gastroscopy and biopsy revealed a large hemorrhagic ulcerated carcinoma located in the antrum, gastric angle, and lower part of gastric body. Abdominal CT indicated an infiltrative ulcerated carcinoma with perigastric lymph nodes metastasis.

**Interventions:**

After fluid resuscitation, blood transfusion, application of proton pump inhibitors, and Octreotide, the patient recovered gradually. Then, nasojejunal feeding tube was placed for enteral nutrition and tumor exclusion. Subsequently, the patient received 5 cycles of neoadjuvant S-1 plus oxaliplatin regimen, without signs of rebleeding, followed by radical distal gastrectomy.

**Outcomes:**

Pathological examination confirmed that the patient received pathological complete response.

**Lessons:**

This case suggests that neoadjuvant chemotherapy is feasible in selected hemorrhagic gastric cancer patients and tumor exclusion is helpful in reducing rebleeding risk.

## 1. Introduction

Locally advanced gastric cancer (LAGC) can be accompanied by bleeding (melena, hematemesis) due to local tumor progression.^[1]^ Hemorrhagic LAGC can present with anemia or chronic malnutrition, even a life-threatening condition due to hematological instability.^[2,3]^ Although endoscopic therapy, interventional therapy and other non-surgical therapies are options for hemostasis, the risk of rebleeding restricts their usage.^[4]^ Emergency operation is generally performed to remove the hemorrhagic tumor.^[3]^ However, emergency surgery patients suffer from higher risk of postoperative complications and poorer long-term prognosis.^[5,6]^ Neoadjuvant chemotherapy (NACT) has been demonstrated to improve tumor remission rate and oncological prognosis in LAGC patients.^[7]^ Nonetheless, neoadjuvant chemotherapy is rarely applied in hemorrhagic patients in consideration of the risk of rebleeding. Additionally, NACT may cause side reactions such as vomiting which may worsen the situation. Therefore, most surgeons perform emergency surgery for hemorrhagic gastric cancer.^[3]^ In this case, we developed a novel therapeutic strategy for a patient with hemorrhagic LAGC. We placed a nasojejunal tube for enteral nutrition and tumor exclusion before NACT. After 5 cycles of the neoadjuvant S-1 plus oxaliplatin regimen, laparoscopic radical distal gastrectomy was then performed. No significant recurrent bleeding was observed throughout treatment and primary tumor achieved pathological complete response. In comparison to surgery alone without preoperative chemotherapy, this case may offer a more optimal therapeutic schedule for selected hemorrhagic LAGC.

## 2. Case presentation

A 58-year-old pale and diaphoretic man was presented to the emergency department with abdominal pain and tachycardia (107/minute). He mentioned a ten-day history of melena with dull epigastric pain, dizziness and fatigue, but didn’t seek treatment. He denied using NSAIDs, consuming alcohol or nicotine, or having a history of gastrointestinal bleeding or other conditions. There were no gastrointestinal disorders or cancers in his family history.

**Figure 1.**
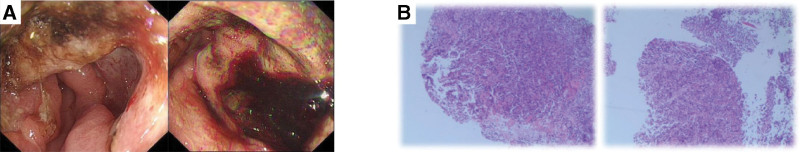
(A) Gastroscopy showed a large ulcerated irregular-shaped elevated lesion covered with a quantity of blood clots. (B) Histologic examination of biopsies showed poorly differentiated adenocarcinoma.

**Figure 2.**
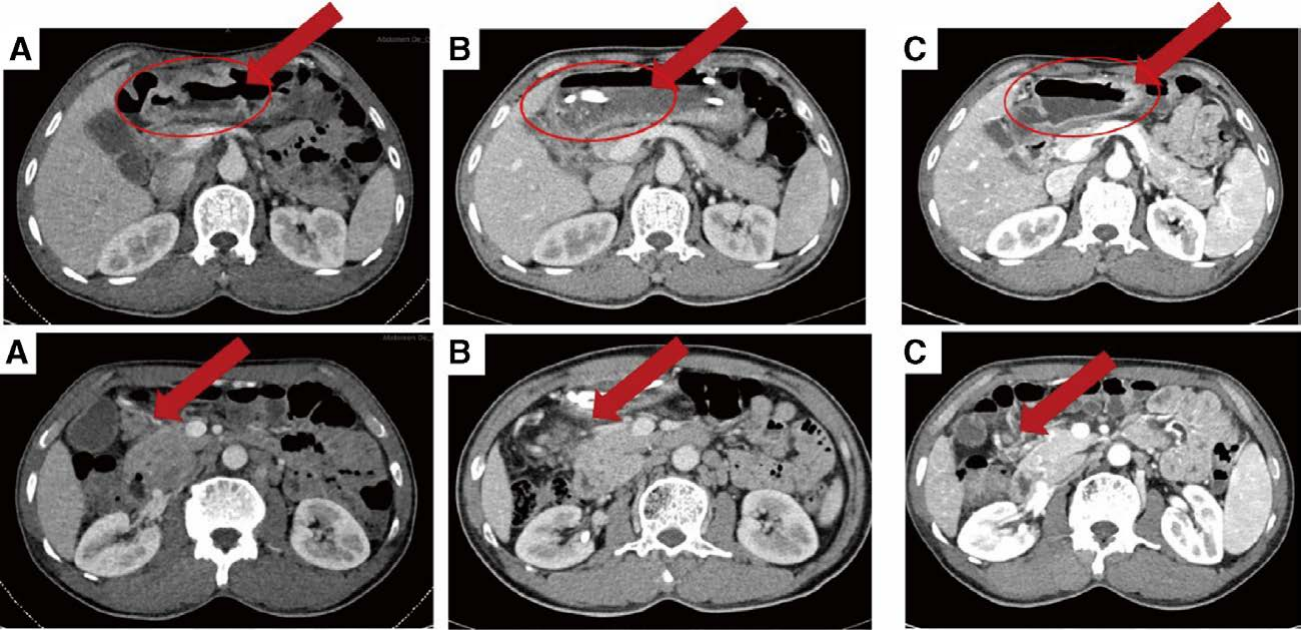
(A) Enhanced CT of abdomen at admission showed irregular thickening of the gastric wall and a 2.2cm*2.0cm enlarged lymph. (B)&(C) Enhanced CT with dynamically review during neoadjuvant chemotherapy revealed the regression of the lesion.

Physical examination revealed pale conjunctiva, tenderness in the upper abdomen, with blood pressure of 99/56 mm Hg, and heart rate of 107/minute. Red blood cell (RBC) count was 2.98*10^^12^/L, hemoglobin (Hb) was 7.2 g/dL, hematocrit (HCT) was 23.7% (Table 1), and blood urine nitrogen (BUN) was 8.4 mmol/L according to preliminary laboratory investigations. Gastroscopy was performed and showed poor gastric peristalsis and rigidity of gastric wall. A large ulcerated irregular-shaped elevated lesion covered with a quantity of blood clots was observed in pyloric antrum involving gastric angle and lower part of gastric corpus, and the pyloric canal was evidently congested and swollen (Fig. 1). Histologic examination of biopsies revealed poorly differentiated adenocarcinoma (Fig. 1) and the patient was finally diagnosed with hemorrhagic gastric cancer. His Glasgow Blatchford score (GBS) was 13 and was classified as Forrest IIb on the Forrest classification. Then, enhanced CT revealed irregular thickening of the gastric wall in the lower part of the gastric body and antrum as well as multiple enlarged lymph nodes around the stomach and retroperitoneum (the largest was 2.2cm*2.0cm, located below the gastric antrum) (Fig. 2). These led to the clinical tumor-node-metastasis staging of the patient being determined to be cT_3+_N_+_M_0_.

**Table 1:** Labs on admission and after total neoadjuvant chemotherapy.

Laboratory test (Normal range)	Value †	Value ‡
WBC (3.5–9.5 × 10^9^/L)	6.1	3.8
RBC (4.3–5.8 × 10^12^/L)	2.98	3.22
HGB (13.0–17.5 g/dL)	7.2	9.3
HCT (40.0–50.0 %)	23.7	29.9
PLT (125–350 × 10^9^/L)	209	155
Albumin (4.0–5.5 g/dL)	2.8	3.3
ALT (9.0–50.0 U/L)	14	25
AST (15.0–40.0 U/L)	15	36
BUN (2.1–7.1 mmol/L)	8.4	6.6

ALT = alanine aminotransferase, AST = aspartate aminotransferase, BUN = blood urea nitrogen, HCT = hematocrit, HGB = hemoglobin, PLT = platelet, RBC = red blood cells, WBC = white blood cells.

† Initial labs on admission;

‡ Labs after 5 cycles of neoadjuvant chemotherapy.

**Figure 3.**
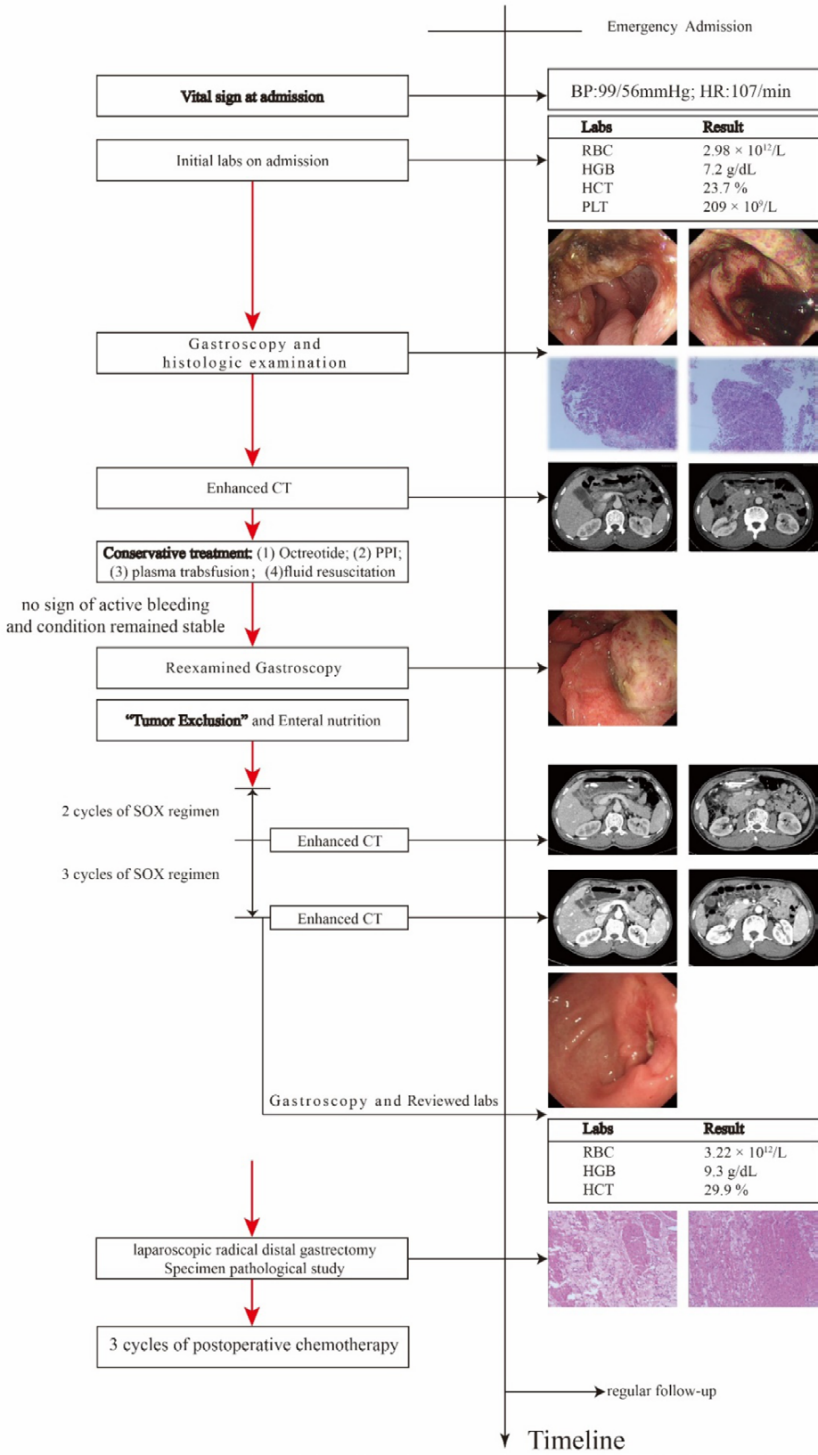
Timeline from emergency to follow-up.

Patients with bleeding gastric cancer usually require emergency surgical resection; nevertheless, patients who undergo emergency surgery suffer more postoperative complications and worse long-term prognosis.^[5,6]^ Considering that the patient had no signs of hemodynamic instability, it was decided to start with conservative treatment (Octreotide, proton pump inhibitor [PPI], fluid resuscitation, and plasma transfusion, etc). Vital signs were dynamically monitored, while hemoglobin and fecal occult blood were daily tested. After conservative treatment, the patient condition remained stable and there was no sign of active bleeding. The lesion was reassessed by gastroscopy 3 days after admission and was classified as Forrest III, indicating a low risk of rebleeding. Then, we placed a nasojejunal tube under gastroscopy for enteral nutrition and “tumor exclusion.” Peptison was pumped daily through the nasojejunal tube to provide nutritional requirements for the patients. Furthermore, the tumor was also free from physical irritation caused by food, lowering the risk of rebleeding. Then, the patient received neoadjuvant chemotherapy with S-1 plus oxaliplatin regimen and was evaluated every 2 cycles. After 5 cycles of NACT, the lesion and lymph nodes were shrunk significantly (Fig. 2). No recurrent bleeding was observed during NACT, and Hb restored to 9.3g/dL (RBC of 3.22 × 1012/L, HCT of 29.9%) (Table 1). Subsequently, the patient underwent laparoscopic radical distal gastrectomy and no residual cancer was present on specimen analysis, which implied pathological complete response. The patient received 3 cycles of postoperative chemotherapy and was under regular follow-up. He was alive 1 year following surgery and had positive spirit and sleep without melena. The timeline from emergency to follow-up is presented in Figure 3.

## 3. Discussion

More than 10% of patients with gastric cancer have bleeding at the first diagnosis, and more than 50% of them are major bleeding.^[8]^ Moreover, bleeding from LAGC affects the subsequent treatment and ultimately result in a poor prognosis.^[9]^ We report a patient who was presented to our hospital with tachycardia and abdominal pain due to hemorrhagic LAGC. He was started on NACT after 9-day of conservative treatment including Octreotide, acid suppression, fluid infusion and tumor exclusion, etc. Subsequently, he underwent radical distal gastrectomy and recovered well.

In patients with acute or severe bleeding (hematemesis or melena), the guidelines recommend to prefer endoscopy first for diagnosis and therapy.^[10,11]^ Although endoscopic hemostasis can control the hemorrhage, it has a high rate (40%–80%) of recurrent bleeding.^[11,12]^ Therefore, reducing the hazard of rebleeding is critical during subsequent treatment in hemodynamically unstable LAGC patients.

Although PPIs have been prescribed to reduce the risk of hemorrhage from gastric cancer, the evidence is not conclusive.^[11]^ A controlled trial observed that PPIs did not significantly reduce the incidence of bleeding from inoperable gastric cancer, but it showed that the application of PPIs in gastric cancer patients receiving chemotherapy reduced tumor bleeding events significantly within the first 4 months.^[13]^ PPIs can raise the pH in the stomach, promote platelet aggregation and reduce platelet disaggregation by degrading activity of pepsin.^[14]^ In addition, an in vitro experiment found that alkaline microenvironment can inhibit the growth of gastric cancer cells and even promote their apoptosis.^[15]^ Accordingly, we employed intravenous and oral PPIs to prevent rebleeding while indwelling nasojejunal tube for “tumor exclusion.” Enteral nutrition was supported by the nasojejunal tube, which prevented the tumor lesion from contacting stimulus like food or drug and also reduced the secretion of gastric acid, further stabilizing and “excluding” the tumor.

Successful hemostasis of hemorrhage from LAGC refers to no evidence of bleeding under the endoscope,^[16]^ and rebleeding is defined as clinical manifestations of upper gastrointestinal bleeding or a decrease in hemoglobin level (>2 g/dL) or as evidence of hemorrhage on endoscopy.^[17]^ The GBS, Rockall (RS), and AIMS65 scoring methods are commonly used to predict recurrent bleeding; GBS is considered to be the most sensitive and specific method, and the risk of bleeding increases with GBS scores ≥ 7.^[18]^ Despite the fact that this patient was hematologically unstable and had a GBS score of 13, no recurrent bleeding was detected during NACT after conservative treatment. This may suggest that if hemostasis is relatively successful, we can aggressively administer neoadjuvant chemotherapy in patients with severe hemorrhagic LAGC and a GBS score of 13 or higher. In addition, the Forrest classification was utilized to assess the risk of rebleeding dynamically, which was practicable in this case. These methods may be useful for predicting the rebleeding in most hemorrhagic gastric cancer, which need to probe further.

The strength of this case is that it highlights a modality of preventing recurrent bleeding during neoadjuvant chemotherapy in patients with hemorrhagic LAGC, including the conduct of “tumor exclusion,” and the utilization of PPIs. Moreover, neoadjuvant chemotherapy can shrink the tumor and reduce the blood supply to the mass,^[19]^ lowering the risk of recurrent bleeding. Although this patient with severe hemorrhagic LAGC had a hematological instability on admission, he was able to receive the whole course of NACT by successfully preventing recurrent hemorrhage, and even achieve pathological complete response. His prognosis is theoretically better than that after an emergent gastrectomy.^[20]^

There were still some limitations in our study. Firstly, EUS and CT scan are 2 routine methods for assessing the depth of tumor invasion (T staging) preoperatively,^[11]^ and CT was preferred in this case. However, we did not have the patient drink enough water for the CT examination due to concerns about rebleeding of lesion, so the diagnostic accuracy for T staging may have been higher if the EUS had been combined. Secondly, although the patient agreed with our therapeutic plan and believed this would prolong his survival, he reported at follow-up that his satisfaction and quality of life during neoadjuvant chemotherapy were poor because he had to be fed through the nasojejunal tube.

## 4. Conclusions

For patients with hemorrhagic LAGC, the employment of PPIs, and tumor exclusion can reduce the risk of tumor rebleeding. If the good hemostatic effect is obtained, neoadjuvant chemotherapy may be acceptable even in hematologically unstable patients with severe hemorrhagic LAGC. The hemorrhagic LAGC patient with a GBS score of 13 may still be managed conservatively for hemostasis without the need for urgent surgery.

## Acknowledgment

The authors would like to appreciate the patient and his family for agreeing to share this case, and we also want to thank Digestive Endoscopy Center of Fujian Provincial Hospital for help.

## Author contributions

**Conceptualization:** Yuhang Zhou, Yuchen Zhou

**Data curation:** Yuhang Zhou, Yuchen Zhou, Xiaojun Lin

**Funding acquisition:** Shengtao Lin, Weihua Li

**Supervision:** Shengtao Lin, Weihua Li

**Writing – original draft:** Yuhang Zhou, Yuchen Zhou

**Writing – review & editing**: Shengtao Lin, Weihua Li
